# Anterior cervical discectomy with fusion in patients with cervical disc degeneration: a prospective outcome study of 258 patients (181 fused with autologous bone graft and 77 fused with a PEEK cage)

**DOI:** 10.1186/1471-2482-10-10

**Published:** 2010-03-21

**Authors:** Bjarne Lied, Paal Andre Roenning, Jarle Sundseth, Eirik Helseth

**Affiliations:** 1 Department of Neurosurgery-Rikshospitalet, Oslo University Hospital, N-0027 Oslo, Norway; 2 Department of Neurosurgery-Ullevål, Oslo University Hospital, N-0407 Oslo, Norway; 3 Faculty of Medicine, University of Oslo, Oslo, Norway

## Abstract

**Background:**

Anterior cervical discectomy with fusion (ACDF) is challenging with respect to both patient selection and choice of surgical procedure. The aim of this study was to evaluate the clinical outcome of ACDF, with respect to both patient selection and choice of surgical procedure: fusion with an autologous iliac crest graft (AICG) versus fusion with an artificial cage made of polyetheretherketone (PEEK).

**Methods:**

This was a non-randomized prospective single-center outcome study of 258 patients who underwent ACDF for cervical disc degeneration (CDD). Fusion was attained with either tricortical AICG or PEEK cages without additional anterior plating, with treatment selected at surgeon's discretion. Radicular pain, neck-pain, headache and patient satisfaction with the treatment were scored using the visual analogue scale (VAS).

**Results:**

The median age was 47.5 (28.3-82.8) years, and 44% of patients were female. 59% had single-level ACDF, 40% had two level ACDF and 1% had three-level ACDF. Of the patients, 181 were fused with AICG and 77 with a PEEK-cage. After surgery, the patients showed a significant reduction in radicular pain (ΔVAS = 3.05), neck pain (ΔVAS = 2.30) and headache (ΔVAS = 0.55). Six months after surgery, 48% of patients had returned to work: however 24% were still receiving workers' compensation.

Using univariate and multivariate analyses we found that high preoperative pain intensity was significantly associated with a decrease in pain intensity after surgery, for all three pain categories. There were no significant correlations between pain relief and the following patient characteristics: fusion method (AICG or PEEK-cage), sex, age, number of levels fused, disc level fused, previous neck surgery (except for neck pain), previous neck trauma, or preoperative symptom duration. Two hundred out of the 256 (78%) patients evaluated the surgical result as successful. Only 27/256 (11%) classified the surgical result as a failure. Patient satisfaction was significantly associated with pain relief after surgery.

**Conclusions:**

ACDF is an effective treatment for radicular pain in selected patients with CDD after six months follow up.

Because of similar clinical outcomes and lack of donor site morbidity when using PEEK, we now prefer fusion with PEEK cage to AICG.

Lengthy symptom duration was not a negative prognostic marker in our patient population.

The number of patients who returned to work 6 months after surgery was lower than expected.

## Background

The vast majority of patients with symptomatic cervical disc degeneration (CDD) respond well to conservative treatment [1]. For nonresponders, surgical treatment using ACDF is an option for selected patients [2]. In the USA, the annual incidence of surgery for CDD is 50- 60 per 100 000 inhabitants [3]. Selection of adequate candidates for ACDF surgery is a continuous challenge. According to the literature, the following are potential positive predictive preoperative markers: intense radicular pain, low disability, young age, soft disc disease in one segmental level, male sex, non-smoker status, presence of a correlation between radiological and clinical findings, good hand strength, good active range of motion in the neck, and no spinal litigations [4,5]. In surgery for lumbar disc degeneration, symptom duration > 6 months is regarded as a negative prognostic factor [6]. This is also reported to be true for ACDF surgery [7]. This is intriguing, as many patients referred to ACDF surgery have symptom duration > 6 months. Is surgery in these patients worthwhile or futile?

The gold standard for ACDF has been fusion with an AICG [8-10]. This is a relatively safe procedure with few complications [11-13]. However, this surgical procedure has been hampered by iliac crest donor site morbidity. This has led to a growing interest in artificial cages made of various materials, including tantalium blocks, titanium, carbonfiber and polyetheretherketone (PEEK), to replace the AICG [12,14-18]. In our hospital since 2004 we have gradually shifted from AICG to PEEK. We found no increase in complications after shifting to fusion with a PEEK cage [11].

We prospectively registered all ACDF patients followed in our department from 2003-2005 and used this information to address the following questions.

1. What improvement in clinical outcomes can be expected after ACDF for CDD with regard to radicular pain, neck pain, headache, and return to work?

2. Did the gradual shift from fusion with AICG to fusion with PEEK cage, in our department during the study period, influence outcomes?

3. Do symptom duration or other preoperative clinical variables correlate with outcome after ACDF for CDD in our series?

## Methods

This was a prospective single-center study of patients who underwent single-, two-, or three- level ACDF for CDD. The study was performed at the Oslo University Hospital-Rikshospitalet in Oslo from 2003 to 2005. All surgeons were asked to participate in a prospective registration of clinical parameters. During this period, 390 patients (total group) were eligible for inclusion and we obtained complete preoperative and follow-up data for 258 patients (66.1%) (study group). Only the 258 patients with complete data sets were included in the analysis.

### Inclusion criteria (1 + 2)

The inclusion criteria were

1. One or more of the following symptoms and signs of CDD:

a. Persistent severe radicular pain not responding to conservative management for three months.

b. Cervical radiculopathy with progressive paresis.

c. Selected cases with myelopathy secondary to cervical spinal canal stenosis that can be adequately decompressed with ACDF.

d. Selected cases with mainly neck pain and headache and less radicular pain.

2. MRI- documented CDD with compression of cervical nerve roots or spinal cord, which most likely explain the clinical symptoms and signs.

### Exclusion criteria

The exclusion criteria were

1. Cervical trauma within the past four weeks.

2. Cervical neoplasia.

3. Ongoing cervical infection.

### Diagnostic work-up

The diagnostic work-up included

1. Clinical and neurological examination.

2. Cervical MRI (cervical CT-myelography was used in one case where MRI was contraindicated due to a permanent pacemaker).

### ACDF

In all patients, we used an anterior approach to the cervical spine with a right-sided skin incision, as originally described by Robinson and Smith [9]. A self-retractor was mounted after verification of the levels of interest using fluoroscopy, (Shadow -line, V. Mueller Neuro/Spine Product, Cardinal Health, San Carlos, CA).

In most patients, an operating microscope was used and the disc was removed with a high-speed drill (Midas Rex, Medtronic, Memphis, TN). Removal of the posterior longitudinal ligament and the final decompression of the nerve roots were performed using small rongeurs. Bilateral nerve root decompression was always performed, even in patients with unilateral symptoms. After the procedure, distraction was applied using the Shadow-line Distraction System (V. Mueller Neuro/Spine Product, Cardinal Health). Fusion was attained with either tricortical AICG or PEEK cages (Cervios, Stratec Medical, Oberdorf, Switzerland), at the discretion of the surgeon. After removal of the Shadow-line distracters, the screw holes were plugged with bone wax (Ethicon, Johnson & Johnson, Somerville, NJ) to prevent postoperative bleeding. Wound drainage was not routinely used. A single dose of cephalothin (30 mg/kg), which was used as infection prophylaxis, was administered 15- 30 min before the skin incision [19-21].

### Iliac crest auto graft

The tricortical AICG was harvested from the right iliac crest. Care was taken to preserve the anterior 2 cm of the iliac crest and the lateral cutaneous femoral nerve. The bone grafts were harvested using an oscillating saw and a graft cutter, and the bone bed was waxed with bone wax (Ethicon, Johnson & Johnson, USA). Wound drainage was not routinely used, and the surrounding soft tissue was infiltrated with 20 ml of bupivacaine after wound closure.

### Postoperative care

The patients were observed in a recovery unit for the first 4 - 6 h after surgery, and were then transferred to the regular neurosurgical ward. All patients were mobilized with a stiff collar within 24 h after surgery. Almost all patients were discharged from our hospital to the referring neurological department 48-72 h after surgery. All patients were encouraged repeatedly to participate in normal activities 6-14 weeks after surgery. A final clinical examination was performed 6 months after surgery in our outpatient clinic.

### Prospective registration of clinical parameters

The parameters registered the day before surgery included age, sex, symptom duration before surgery (months), previous surgery for CDD, previous neck trauma, working status, radicular pain, neck pain, headache, myelopathy (yes/no), and paresis (muscular strength graded according to the Royal Medical Research Council of Great Britain, where 5 is normal strength and 0 is total paralysis in the affected muscle group)[22]. Each of the three pain categories was scored using a VAS, where 0 indicated no pain and 10 represented extreme pain[23].

As the clinical impact of changes in VAS scores less than ± 2 is unclear, we estimated the number of patients that had changes in VAS scores of more than ± 2 for the three pain categories [24,25]. The parameters registered during surgery included: number of levels fused (single-level, two-level, or three-level fusion), level fused (C3/C4, C4/C5, C5/C6, C6/7 or C7/Th1) and fusion type (AICG or PEEKcage). The following parameters were registered at the 6-month follow-up visit in our outpatient clinic: radicular pain, neck pain, headache, myelopathy (a diagnosis of myelopathy required neurological signs of upper motor neuron affection as Babinsky sign, hyperreflexia or increased muscular tone), paresis, working status and patient satisfaction with the surgical treatment. Patient satisfaction was measured using a VAS scale, where a score of 0 indicated that the patient was not at all satisfied with the result of ACDF and a score of 10 indicated that the patient was very satisfied with the surgical outcomes[26,27]. We defined a VAS score ≥ 8 as a success, while a score ≤ 5 was regarded as a failure.

### Surgery-related complications

We have previously published our complications in 390 consecutive ACDF operations, which included 278 patients fused with AICG and 112 patients fused with a PEEK graft [11].

### Database and statistical analyses

For linear regression analysis, we first performed a univariate analysis, followed by multivariate modeling introducing all the variables, in an exploratory fashion. The linearity assumption of the linear regression was checked using a plot of the fitted regression line compared with a locally weighted nonparametric scatterplot of the outcome variable against the predictor. Homoscedasticity was checked by graphing residuals versus predicted and observed values. Finally, normality of residuals was checked using boxplots, histograms, and quantile plots of residuals. Ordinal variables were also checked for linearity using a nested likelihood ratio test.

Some of our variables displayed heteroscedasticity, therefore, we repeated the analyses using both the Huber-White sandwich estimator of variance relaxing the homoscedasticity assumption and bootstrapped regressions with 1.000 repetitions. The results of these analyses were in agreement with the findings of our traditional regression results, which allowed us to take a relaxed stance toward the heteroscedastic findings in some of our models. Standard paired t-tests, chi-squared, and z-tests for proportions were also used. Significance was set at alpha < 0.05. The Stata v10.1 (Stata Corp, Austin, TX) software was used in all analyses.

### Ethics

The Data Protection Officials of the Rikshospitalet approved the study. All patients gave signed informed consent for entry of the data into the database and for the subsequent prospective study.

## Results

### Baseline clinical characteristics

Of the 390 patients eligible for inclusion in this study (total group), we obtained complete preoperative- and follow-up data for 258 patients (66.1%) (study group). The patient characteristics for both groups are included in Table 1. No significant differences were found between the groups with respect to baseline clinical characteristics. Only the 258 patients with complete data sets were included in the analyses.

**Table 1:** Patient characteristics.

ACDF	Study group N = 258	Total group N = 390	p-value
Females - no of patients (%)	114 (44)	178 (46)	0.72^prop^
Median age (range) - (years)	47.5 (28.3-82.8)	47.7 (26.9- 82.8)	0.73

Levels per procedure - no of patients (%)			
One level	152 (59)	240 (61)	0.50
Two levels	104 (40)	148 (38)	0.55
Three levels	2 (1)	2 (1)	0.68 0.75^χ2^

Level - no of patients (%)			
C3/C4	7 (3)	7 (2)	0.43
C4/C5	38 (15)	56 (14)	0.90
C5/C6	182 (71)	266 (68)	0.53
C6/C7	137 (53)	207 (53)	0.99
C7/Th1	2 (1)	6 (2)	0.39 0.85^χ2^

Method of fusion - no of patients (%)			
Autologous bone graft	181 (70)	278 (71)	0.76
PEEK* cage	77 (30)	112 (29)	0.76 0.76^χ2^

Symptoms - no of patients (%)			
Radiculopathy	206 (80)	309 (79)	0.85
Radiculopathy and myelopathy	36 (14)	50 (13)	0.86
Myelopathy	9 (3)	18 (5)	0.48
No radiculopathy or myelopathy	7 (3)	13 (3)	0.66

Previous ACDF - no of patients (%)	11 (4)	18 (5)	0.83

Previous neck trauma - no of patients (%)	23 (9)	26 (7)	0.29

*Polyetheretherketone

### Pain relief after surgery

We found a significant reduction in radicular pain, neck pain, and headache after surgery in the study group (Table 2). The reduction was most pronounced for radicular pain and neck pain. Using univariate and multivariate analyses we found that high preoperative pain intensity was significantly associated with a decrease in pain intensity after surgery, for all three pain categories. There were no significant correlations between pain relief and the following patients characteristics: sex, age, number of levels fused, disc level fused, fusion method (AICG versus PEEK-cage), previous neck surgery (except for neck pain), previous neck trauma, or preoperative symptom duration (Table 3). As the clinical impact of changes in VAS scores less than ± 2 is unclear, we estimated the number of patients that had changes in VAS scores of more than ± 2 for the three pain categories (Table 4). Radicular pain improved ≥ 2 VAS points in 64% of the patients, while 6% of patients experienced a worsening of VAS scor ≤ -2. Neck pain improved ≥ 2 VAS points in 55% of the patients, while 10% of patients experienced a worsening of VAS score ≤ -2. Headache improved ≥ 2 VAS points in 31% of the patients, while 16% of patients experienced a worsening of VAS score ≤ -2.

**Table 2:** Intensity of pain measured using the VAS scale before surgery (preop) and 6 months after surgery (postop).

			Paired differences			
				95% Confidence interval		
Paired samples	N	Mean	Mean	Lower	Upper	Sig. (two-tailed)
Preop radicular pain	258	7.47				
			3.05	2.65	3.45	0.000
Postop radicular pain	258	4.42				
Preop neck pain	255	6.45				
			2.30	1.90	2.71	0.000
Postop neck pain	255	4.15				
Preop headache	254	3.63				
			0.55	0.22	0.89	0.001
Postop headache	254	3.08				

(Paired sample t-test).

**Table 3:** Univariate and multivariate Cox regression model of potential predictors of outcome.

	Delta radicular pain		Delta neck pain		Delta headache	
	Univariate	Multivariate	Univariate	Multivariate	Univariate	Multivariate
Sex	0.25 [-0.56, 1.05]	0.031 [-0.73, 0.79]	0.40 [-0.41,1.22]	0.18 [-0.49, 0.85]	0.34 [-0.34, 1.01]	0.15 [-0.45,0.74]
						
Age	-0.035 [-0.08, 0.01]	0 .	0.0095 [-0.03, 0.05]	0 .	-0.000059 [-0.04, 0.04]	0 .
						
Type of fusion	0.0077 [-0.87, 0.88]	0.21 [-0.61, 1.04]	0.096 [-0.78, 0.98]	-0.069 [-0.79, 0.66]	-0.21 [-0.94, 0.52]	-0.040 [-0.69, 0.61]
						
No of levels	0.40 [-0.38, 1.19]	0.35 [-0.49, 1.18]	0.23 [-0.56, 1.02]	0.14 [-0.60, 0.87]	0.32 [-0.34, 0.98]	0.37 [-0.28, 1.03]
						
Level	0.088 [-0.46, 0.64]	0.055 [-0.53, 0.64]	0.35 [-0.20, 0.91]	0.068 [-0.45, 0.58]	0.019 [-0.44, 0.48]	-0.18 [-0.64, 0.28]
						
Previous symptom duration	-0.0019 [-0.01, 0.01]	-0.0016 [-0.01, 0.01]	0.0028 [-0.00, 0.01]	-0.00088 [-0.01, 0.01]	0.0018 [-0.00, 0.01]	-0.0014 [-0.01, 0.00]
						
Previous neck surgery	-0.62 [-2.59, 1.36]	-0.50 [-2.37, 1.36]	-1.75 [-3.73, 0.23]	-1.82* [-3.45, -0.18]	-1.31 [-3.03, 0.41]	-1.02 [-2.54, 0.51]
						
Previous neck trauma	0.24 [-1.17, 1.64]	-0.098 [-1.42, 1.23]	-0.58 [-1.99, 0.83]	-0.37 [-1.53, 0.79]	0.011 [-1.16, 1.18]	-0.53 [-1.56,0.51]
						
Preop radicular pain	0.60*** [0.44, 0.76]	0.63*** [0.45, 0.81]	0.31*** [0.13, 0.48]	-0.030 [-0.19, 0.13]	0.065 [-0.08, 0.21]	-0.021 [-0.16, 0.12]
						
Preop neck pain	0.14 [-0.00, 0.28]	-0.075 [-0.23, 0.08]	0.66*** [0.54, 0.78]	0.75*** [0.62, 0.89]	0.16** [0.04, 0.28]	-0.035 [-0.16, 0.09]
						
Preop headache	0.078 [-0.07,0.22]	0.041 [-0.11, 0.19]	0.087 [-0.06, 0.23]	-0.22** [-0.35, -0.09]	0.52*** [0.41, 0.62]	0.54*** [0.42, 0.66]
						

Observations		256		255		252
						

Adjusted *R*^2^		0.150		0.350		0.261

95% confidence intervals are shown in brackets

*p *< 0.05, ** *p *< 0.01, *** *p *< 0.00

Outcome variables are the paired differences between preoperative and postoperative pain VAS scores (delta radicular pain, delta neck pain and delta headache).

**Table 4:** Number of patients with changes in VAS scores of more than ± 2 for the three pain categories, radicular pain, neck pain and headache, separately.

	Delta VAS = preoperative - postoperative		
**Pain category**	**Worsened (≤ -2VAS)**	**Unchanged**	**Improved (≥ 2 VAS)**
Radicular pain	16 (6.2%)	78 (30.2%)	164 (63.6%)
Neck pain	26 (10.1%)	91 (35.3%)	141 (54.7%)
Headache	41 (15.9%)	136 (52.7%)	81 (31.4%)

### Paresis

One hundred and fifty-one of the 249 (61%) patients had normal muscular strength at the time of surgery. At follow-up, 233/249 (94%) patients had normal muscular strength.

### Myelopathy

Of the 45 patients with clinical evident myelopathy at the time of surgery, only 16 (35.6%) had persistent myelopathy 6 months after surgery.

### Working status

At the onset of symptomatic CDD, 80% of patients were employed fulltime, 3% received workers ' compensation for CDD, 4% received workers ' compensation for reasons other than CDD, 8% received a disability pension, and 5% were students, housewives, retired, or unemployed. At the time of surgery, 66% of patients had received workers ' compensation for 1 month or more. The median sick leave before surgery was 5.0 (0-150) months. Six months after surgery, 48% of patients had returned to work: however 24% were still receiving workers ' compensation. The percentage of patients receiving a disability pension increased from 8%, before the onset of symptomatic CDD, to 21% 6 months postoperatively. The increase in the number of patients receiving a disability pension was, related to CDD in all cases but one.

### Patient satisfaction

At the 6-month postoperative control, all patients were asked to score their satisfaction with the surgical result using a VAS scale. The mean reported VAS score was 8.42, and 200/256 (78%) patients reported a score > 8 (success). Only 27/256 (11%) patients reported a VAS score < 5, which indicate that the operation did not fulfil their expectations (failure). Patient satisfaction was then correlated to other measures of surgical outcome at 6 months (Table 5). Patient satisfaction was significantly associated with pain relief after surgery.

**Table 5:** Patient satisfaction 6-months after surgery correlated with selected preoperative variables and other measures of surgical outcome at 6 months.

	Patient satisfaction
Sex	-0.19 [-0.85, 0.46]
	
Age	-0.031 [-0.07, 0.00]
	
Previous symptom duration	0.00014 [-0.01, 0.01]
	
Delta radiculopathy	0.33*** [0.24, 0.42]
	
Delta neck pain	0.22*** [0.12, 0.31]
	
Delta headache	0.20** [0.08, 0.31]
	

Observations	

(Linear univariate regression analysis).

95% confidence intervals are shown in brackets

* *p *< 0.05, ** *p *< 0.01, *** *p *< 0.001

## Discussion

### Symptom relief after ACDF

The effectiveness of ACDF in relieving radicular pain secondary to CDD is well documented in both long- and short-term follow-up studies [12,15,28-30]. However, the effectiveness of ACDF in relieving neck pain and headache secondary to CDD remains unclear. In this prospective study of 258 patients, we confirmed the beneficial effect of ACDF on radicular pain. The patients also reported a significant improvement in their neck pain and headache. The reduction in headache, although significant, was only by 0.55 points on the VAS scale, in contrast to the changes observed for radicular and neck pain (VAS score variation of 3.05 and 2.30, respectively). The clinical impact of changes in VAS scores < 2 is unclear. An improvement in VAS score ≥ 2 was observed in 64%, 55%, and 31% of patients, for radicular pain, neck pain, and headache, respectively. Almost all patients in our series had radicular pain: therefore, our cohort cannot be used to answer the question concerning the effect of ACDF on neck pain or discogenic headache in patients with mild or no radicular pain. Schofferman et al. published a series of nine patients that allowed them to conclude that ACDF is an effective treatment for discogenic headache [31]. In a recent publication, Laimi et al. reported a low probability of association between headache and CDD [32]. We remain reluctant to offer ACDF to patients with dominating neck pain or headache who have little or no radicular pain.

### Working status

The percentage of patients that returned to work within 6 months of surgery was lower than expected [28]. The most likely explanations for this result are symptom persistence, passive approach with respect to motivating the patient to return to work, and that surgery in some patients was regarded as the last necessary step for the collection of a permanent disability pension. More effort is required to assist the return of patients to the workplace as early as possible. Bhandari et al. reported that 28% of their patient cohort had not returned to work one year after cervical discectomy [33]. These authors found that long preoperative sick leave and persistent postoperative neck pain were associated with not returning to work after surgery. Age and disability claims also influenced the rates of return to work. Steinmetz et al. have studied return to work in a cohort of patients who had workers compensation as their primary insurance. They found 42% return to work 6 months after ACDF and 55% return to work 6 months after cervical disc arthroplasty [34].

### Patient satisfaction

Two hundred out of the 256 (78%) patients evaluated the surgical result as successful. Only 27/256 (11%) patients classified the surgical result as a failure. A 78% success rate after surgery for CDD must be regarded as acceptable. Patient satisfaction is often evaluated using the Odom Criteria [35]. This said, the VAS scale is an accepted tool to evaluate patient satisfaction[26].

### Prognostic factors

In our series, we found a significant correlation between high preoperative pain intensity and decrease in pain intensity after surgery, for all three pain categories. We found no significant correlation between symptom reduction after ACDF and sex, age, number of levels fused, disc level fused, fusion method (AICG or PEEK), previous neck surgery (except for neck pain), previous neck trauma or preoperative symptom duration. The most likely explanation for the lack of identification of prognostic factors is that we selected the best candidates for surgery based on previous knowledge [4,5]. However we were surprised by the fact that symptom duration failed to influence the final surgery outcome, as many of our patients had a rather long preoperative symptom duration. This finding is in contrast with earlier reports on symptomatic CDD and herniated lumbar disc with sciatica [6,7]. An unfavorable postoperative outcome was reported in cases where symptom duration exceeded 6 months in patients treated for herniated lumbar disc and sciatica [6]. Our data suggest that a lengthy duration of symptoms does not influence outcomes.

### Fusion with PEEK cage versus AICG

Anterior cervical decompression and fusion with autologous bone graft has been the standard treatment for CDD for more than 50 years [9]. In recent years, many surgeons have replaced autologous bone grafting with an artificial cage and they report equivalent clinical outcomes after this shift in surgical procedure [12,14-17]. Our study confirmed the results of these previous studies. We found no significant differences between the type of fusion in relation to reduction of radicular pain, neck pain, or headache. We have reported the presence of similar complication rates for patients fused with a PEEK cage or with AICG, with the exeption of the absence of donor site morbidity in patients fused with a PEEK cage [11]. The absence of donor site morbidity, the shorter operation time, and the equivalent clinical results associated with the use of PEEK cages lead us to prefer this type of fusion to AICG.

### Optimal surgical procedure for CDD

There is no clear consensus regarding the optimal surgical procedure for CDD [2,36-38]. Which procedure provides the best clinical outcomes: anterior cervical discectomy alone (ACD), ACDF, discectomy with intervertebral fusion and instrumentation (ACDFI), or cervical arthroplasty? A recent prospective randomized study comparing ACD, ACDF and ACDFI in patients with CDD showed no significant differences in clinical outcomes at the 2 year follow up[37]. However, patients operated with ACD had a higher rate of segmental kyphosis than patients operated with ACDF or ACDFI. Some authors report lesser graft dislocations and graft collapse and higher fusion rates after ACDFI compared with ACDF [13,39,39-42]. On the other hand, the complication rate after ACDFI is somewhat higher compared with ACDF [12,13,41]. ACD, ACDF and ACDFI reduce segmental motion and cause heightened stress on the discs below and above the fusion, which in turn may induce adjacent-level degeneration [43-46]. The main arguments in favor of cervical arthroplasty are the preservation of segmental motion and a lower risk of adjacent-level disc degeneration. The results of randomized, controlled clinical trials comparing cervical disc arthroplasty with ACDF are now emerging [45,47-54]. The follow-up times in the arthroplasty studies are relatively short, however there is a tendency for slightly improved outcomes after cervical prosthesis compared with ACDF. Our routine procedure has so far been ACDF, (both single-level and two-level ACDF). Based on the current literature, we see no reason to change this strategy at this time, although we accept that ACD, ACDFI, and prosthesis probably provide similar clinical outcomes. If the long-term clinical outcome of cervical arthroplasty is demonstrated to be superior to ACDF, we will change our treatment strategy.

### Limitations of the study

• The patients were not randomized to fusion with either AICG or PEEK-cage. The type of fusion was in each case decided by the surgeon. This may cause a bias in the material.

• Ideally the outcome after ACDF for CDD should have been compared with an equivalent group managed with conservative measures.

• The follow-up evaluation was done by the surgeons and not an independent investigator, this may have influenced the final result.

## Conclusions

• ACDF is an effective treatment for radicular pain in selected patients with CDD (patients evaluated 6 months after surgery).

• Because of similar clinical outcome and lack of donor site morbidity when using PEEK, we now prefer fusion with PEEK-cage to AICG.

• Lengthy symptom duration was not a negative prognostic marker in our patient population.

• The number of patients who returned to work 6 months after surgery was lower than expected.

## Competing interests

The authors did not receive financial support in conjunction with the generation of this article. The authors have no personal or institutional financial interest in drugs, materials, or devices described in this article.

## Authors' contributions

BL; libb@ulleval.no; Data collection, statistics, data analysis, discussion and writing. PAR; rnpl@uus.no; Statistics, discussion and writing. JS: jarle.sundseth@rikshospitalet.no; Data collection and discussion. EH; heej@uus.no; Data collection, statistics, data analysis, discussion and writing. All authors have read and approved the final manuscript.

## Pre-publication history

The pre-publication history for this paper can be accessed here:

http://www.biomedcentral.com/1471-2482/10/10/prepub
